# Perceived work environment and patient-centered behavior: A study of selected district hospitals in the central region of Ghana

**DOI:** 10.1371/journal.pone.0244726

**Published:** 2021-01-25

**Authors:** Gordon Abekah-Nkrumah, Jacqueline Nkrumah

**Affiliations:** 1 Department of Public Administration and Health Services Management, University of Ghana Business School, Accra, Greater Accra Region, Ghana; 2 Faculty of Science Education, Department of Health Administration and Education, University of Education, Winneba, Central Region, Ghana; Universiteit van Amsterdam, NETHERLANDS

## Abstract

**Introduction:**

Quality work environment has been established as a marker of employee value creation. A plethora of qualitative evidence suggested that sustained focus on employee satisfaction through changes in the work environment, communication of patient-centered care strategic vision, management of staff workload, and workplace social support are factors that stimulate Patient-centered care. Yet, it seems that the effect of work environment on the patient-centered behavior of hospital employees has not been statistically estimated, and it is unclear which of the elements of the work environment best predict patient-centered behavior.

**Methods:**

Using a survey design and quantitative methods to gather and analyze data, a sample of 179 respondents from three district hospitals were included in the study using a multi-stage proportional sampling technique. Data were collected using self-administered Likert item questionnaires. Simple linear regression was used to estimate the influence of work environment elements on patient-centered behavior. Stepwise multiple regression was used to determine the best predictors of patient-centered behavior of hospital employees.

**Results:**

Perceived internal communication of patient-centered care strategies (β = 0.23; P<0.001), supervisor support (β = 0.31; P<0.001), coworker support (β = 0.50; P<0.001), and working conditions (β = 0.18; P<0.013) had a positive significant effect on patient-centered behavior of employees. Good predictors of employees’ patient-centered behavior were perceived coworker support (β = 0.51; P<0.001) and job characteristics (β = 0.16; P<0.01).

**Conclusion:**

The work environment of hospital employees significantly affects their patient-centered behavior. Co-worker support and job characteristics were the best predictors of the patient-centered behavior of hospital employees. Hospitals Managers seeking to improve patient-centered behavior through employee value creation may consider improved job characteristics in combination with workplace social support and or communication of PCC strategies and goals.

## Introduction

Quality work environment has been suggested as a marker of employee value creation in organizations [1]. In the healthcare quality literature, it is argued that fostering a quality work environment promotes healthcare professionals’ commitment to patient-centered care (PCC) [2]. Studies have suggested sustained focus on employee satisfaction through changes in the work environment, communication of PCC strategic vision, practicable staff workload, and workplace social support as factors that stimulate PCC [3–5]. For instance, Sheller [2], stresses the importance of nurturing an environment that values and treats professionals in the same manner organizational leaders expect employees to care for patients and families to the success of PCC. Lloyd, Elkins, and Innes [5] have also specified staff workload, positive staff relationships, and support as factors that affect centered care.

However, these relationships were established through qualitative analyses, and it seems that a minimal attempt has been made to statistically estimate the effect of the elements of work environment on the patient-centered behavior of hospital employees. Given that the work environment of hospital employees has several elements, estimating the effect of the different dimensions of work environment on patient-centered behavior of employees will be key to establishing specific dimensions that better predict patient-centered behavior of hospital employees. Such information is essential to the development of interventions at the organizational-level to facilitate patient-centered behavior, and to improve the Patient-centered experience of care. It is important to note that the vast evidence on the influence of perceived work environment and performance in healthcare settings focus on clinical staff and nurses in particular to the exclusion of the other categories of employees. The focus of these studies has been to estimate the effect of work environment on nurses’ satisfaction and motivation, patient safety, patient satisfaction, and patient experience [6–8], and not on patient-centered behavior of the different categories of hospital employees. Thus, the current study seeks to contribute to the literature on value-based healthcare, employee value creation, and PCC by statistically estimating the effect of perceived work environment on patient-centered behavior of hospital employees in three district hospitals from the Central Region of Ghana.

### Conceptual framework

The study used B. F. Skinner’s reinforcement theory of motivation to explain the relationship between perceived work environment and patient-centered behavior of hospital employees [9]. Reinforcement relates to the use of stimuli to elicit desired behaviors with different occurrences and schedules. It underscores the effect of environmental factors on behavior and has three continuous parts, namely, stimulus, response, and result [10]. The theory prescribes four methods by which behavior can be affected, including, positive reinforcement, negative reinforcement, extinction, and punishment [10]. Stimulus generates a response in behavior, which consequently leads to results. In this study, quality work environment is considered a pleasant stimulus and a positive reinforcement that can elicit patient-centered behavior. Thus, providing quality work environment can serve as a positive reinforcement that will produce patient-centered behavior among hospital employees. We used the stimulus and response aspects of positive reinforcement to explain the relationship between quality work environment (stimulus) and patient-centered behavior (response). Patient-centered behavior is thought of as a response that could lead to patient-centered experience of care (result) [11].

**Fig 1:**
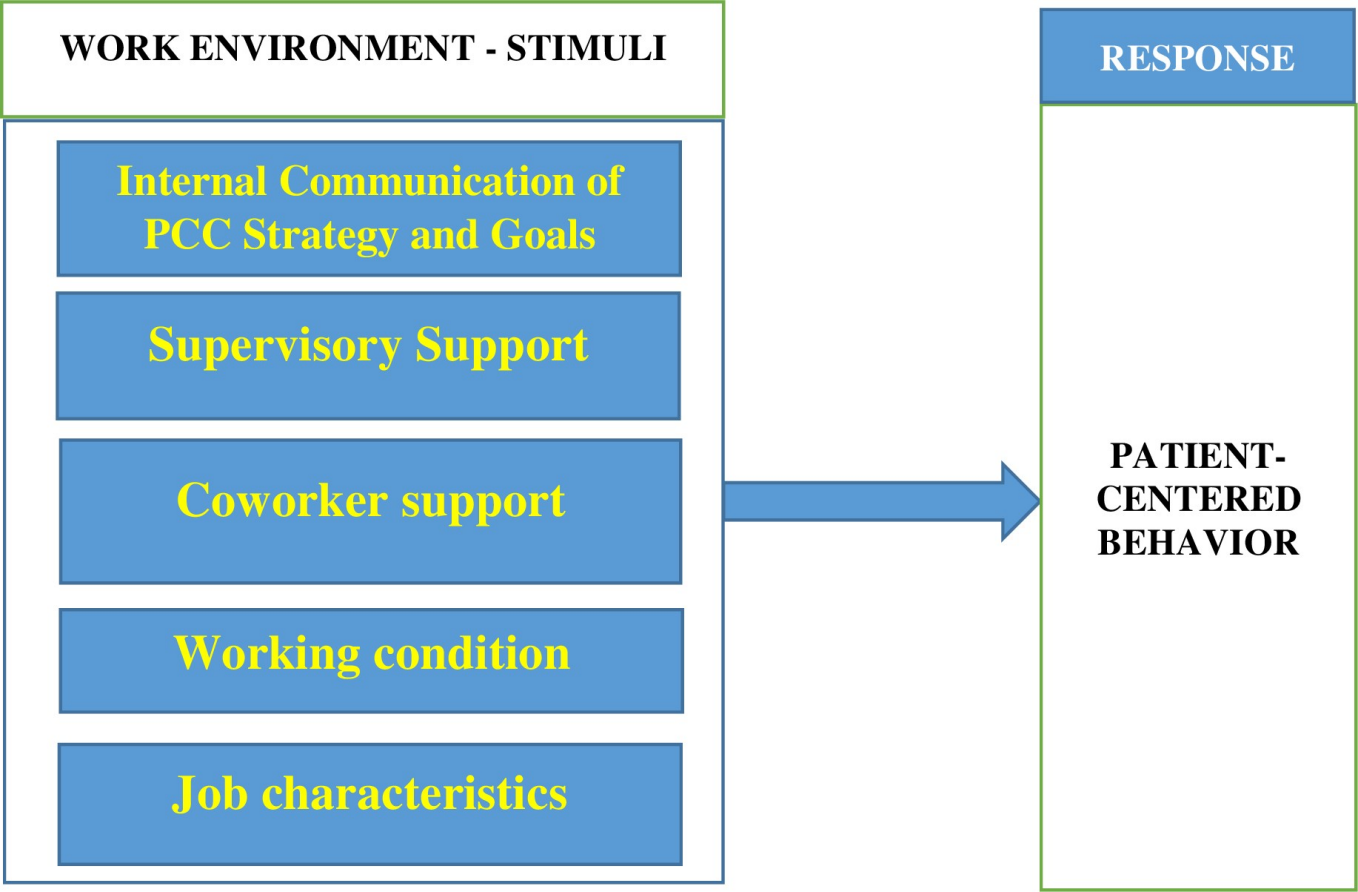
Conceptual framework of the work environment and patient-centered behavior. Source: constructed by authors based on literature review.

Fig 1. provides a conceptual framework of hospital employees’ perception of the work environment and patient-centered behavior to explore the effect of the work environment on hospital employees’ patient-centered behavior.

### Patient-centered behavior

PCC has been defined by several scholars [2, 12, 13], with each definition emphasizing specific elements as the embodiment of PCC. Patient-centered behavior in this study was assessed based on the attributes of PCC developed by Dale Shaller [2]. Shaller’s work is a synthesis of PCC definitions proposed by several authors. It underscores education and shared knowledge, the involvement of family and friends, collaboration and team management, sensitivity to medical and spiritual dimensions, respect for patients' needs, and preferences, and free flow and accessibility of information as attributes of PCC.

### Empirical literature on work environment and employee performance

The health service literature has identified quality of work environment [4, 5] as an important precursor of employee commitment to PCC. Also, in the organizational research literature, substantial evidence exists on the relationship between work environment and employees’ performance [14–16]. Studies have established that a supportive work environment affects employee satisfaction and motivation, which in turn influences performance [17]. In the paragraphs that follow, we discuss empirical findings of the effect of work environment on employee performance.

### Working conditions and job characteristics

Working conditions are a major aspect of the work environment of hospital employees. According to the International Labor Organization [18], working conditions are concerned with the working period, remuneration, physical conditions, and mental demands of a job. Working conditions and job characteristics have been identified as important factors of employee performance. Literature suggests that access to modernized offices, lighting, and ventilation are factors that influence staff motivation, job satisfaction, and patient safety among nurses [19, 20]. In addition to working conditions, it is argued that job characteristics influence employee performance. Studies have found autonomy, Job variety, and task identity as strong predictors of performance among nurses [21, 22]. Workload, job security, and shift work are other aspects of job characteristics that influence employee performance [23]. Studies among correctional staff suggest that, job involvement, feedback, perceived dangerousness of job, and role stress are significant predictors of job performance and stress [24]. Unfortunately, literature on the effect of working conditions and job characteristics on patient-centered behavior of hospital employees is limited [24, 25]. Based on the above discussions, we hypothesize the relationship between perceived working conditions, job characteristics, and patient-centered behavior as follows:

**H**_**1**_: Hospital employees’ perception of working conditions positively influences their patient-centered behavior.
**H**_**2**_: Hospital employees’ perception of job characteristics is positively related to their patient-centered behavior.

### Internal communication

Internal communication in organization promotes free flow of information, collaboration among employees, and affects employee performance. Even though internal communication of PCC goals and strategies have been established as a facilitator of PCC in healthcare organizations [6], the extent to which it influences patient-centered behavior of employees is not well known in the health service literature. Studies that have investigated internal communication and performance have reported a direct and indirect relationship between the two variables [6, 26, 27]. In performance management research, job satisfaction and motivation have been proposed as a mediating variable between organizational communication and employee performance [6, 26]. Also, it has been found that internal communication is an antecedent to employee performance [26]. Studies among nurses have found a positive relationship between nurses’ performance score and conversation scores for the planning, evaluation, assessment, and implementation of nursing care plans [28, 29]. Thus, we hypothesize the relationship between perceived communication of PCC strategies and goals and patience-centered behavior as follows:

**H**_**3**_: Hospital employees’ perception of communication of PCC strategies and goals positively influences their patient-centered behavior.

### Social support

Social support is one of the dimensions of organizational support. It is a major job resource that can be obtained within the workplace and outside of the workplace [30]. Workplace social support comes in two main forms, namely, supervisory support and co-worker support [31, 32]. Employees expect organizations and supervisors to be supportive, promote their welfare, and value their contributions to the achievement of their unit’s objective [31]. Further, employees have expectations about work-related support from co-workers. The general expectation is that their relationship with co-workers will aid and assist them in performing their duties [32]. Social support improves employees’ health and wellbeing [33]. In a study among nurses, a significant relationship was found between perceived co-worker support and nurses’ performance, as well as a positive effect of perceived supervisory and co-worker support on nurses’ work engagement [34]. It has also been found that perceived relationship with supervisors is effective in solving work and non-work-related issues among nurses [35]. The foregoing evidence emphasizes the role of workplace social support on employee performance. Yet, its effect on patient-centered behavior has received minimal attention. Following from the above discussion, we test the following hypotheses:

**H**_**4**_: Hospital employees’ perception of supervisory support positively influences their patient-centered behavior.
**H**_**5**_: Hospital employees’ perception of co-worker support positively influences their patient-centered behavior.

## Materials and methods

### Study institutions

Data for the study was obtained from three district hospitals in the Central Region of Ghana. The Central Region has 20 districts with each district having one district hospital which serves as a first-level referral hospital. St. Luke Catholic Hospital, Effutu Municipal Hospital, and Ajumako District Hospitals were selected from Gomoa West District, Effutu Municipal, and Ajumako-Anyan Assiam District respectively. The three districts have an estimated population of 153,570, 79,411, and 160,813 respectively. Effutu Municipal Hospital is a 143-bed capacity hospital and has 344 staff members. St. Luke Catholic Hospital-Apam has 103 beds and 235 staff. Ajumako District Hospital is a 55-bed hospital with 184 staff. Each of the three hospitals is managed by a management team made up of the Health Service Administrator, Medical Superintendent, Nurse Administrator, and other key functional heads.

### Study design, sampling, and research instrument

The study employed a survey design and quantitative methods to collect and analyze data. A survey design was adopted to enable authors to collect data on the patient-centered behavior of employees and their perception of the workplace environment using a self-reported questionnaire. The study population included district hospitals and hospital employees in the Central Region of Ghana. Multi-stage sampling was used to select hospitals and employees. In the first stage, three out of the 20 districts in the Central Region were conveniently sampled for inclusion in the study based on proximity and lower cost. The second stage involved a purposive selection of three district hospitals from the three districts selected in the first stage based on size (30-bed capacity or more) and governance structure as spelled out in section 31 of the Ghana Health Service and Teaching Hospitals Act (Act 525 of 1996) [36]. The third stage involved stratified proportional sampling of clinical and support service employees from the three district hospitals selected at the second stage. Two hundred and seventy (270) hospital employees were drawn proportionately to the staff strength of the hospitals in the study using the formula n_h_ = (n)N_h_/N [37]. Where n = total sample size (270), N_h_ = stratum size (the staff strength of each hospital), and N = the total employee population of the three hospitals (763). Stratified proportional sampling was used to ensure that the number of employees drawn from each hospital is proportional to the staff strength of that hospital. One hundred and thirty-six (136) hospital employees were selected from Effutu Municipal Hospital, 41 employees from Ajumako District Hospital, and 93 employees from St. Luke Hospital.

Sampling frames of hospital employees were obtained from the selected district hospitals and unique numbers were generated for each employee in the sampling frame using Microsoft Excel. Employees from each hospital were then randomly selected based on their unique numbers using Microsoft Excel. To be included, a participant was supposed to be an employee of the three selected hospitals and must have practice experience of not less than one year. Hospital managers including unit heads and interns were excluded from the study. This is because the focus of the study was to investigate the patient-centered behavior of employees excluding managers and supervisors.

Data for the study was collected with the use of self-administering questionnaires. The data collection instrument measured one dependent variable, which is patient-centered behavior, and five independent variables, namely, perceived internal communication of PCC strategies and goals, supervisory support, co-worker support, working conditions, and job characteristics. The questionnaire was prepared based on an extensive literature review on PCC and the elements of work environment [2, 4, 6, 12, 13, 32, 34, 38, 39, 45]. These studies have measured PCC in the healthcare setting as well as work environment and performance in healthcare non-healthcare organizations. The key attributes of internal communication, workplace social support, working conditions, job characteristics, and PCC as used in the studies cited above were found useful and were adapted to develop the questionnaire items for this study. Questionnaires were checked for content validity by experts in health service management and senior researchers. Questionnaires were pretested among 30 hospital employees at the Central Regional Hospital (Trauma and Specialist Hospital, Winneba) and the results were used to revise the questionnaire before data collection.

Questionnaires were checked for reliability. The dependent variable, Patient-centered behavior was measured on a 5-point Likert scale (never = 1 to always = 5) using 8-items, α = 0.82. The independent variables; perceived internal communication of PCC strategies and goals was measured on a 5-point Likert scale (never = 1 to always = 5), using 7-items, α = 0.88. Perceived supervisory support had 11 items measured on a 5-point Likert scale (strongly disagree = 1 to strongly agree = 5), with α = 0.89. Perception of co-worker support had 11 items measured on 5- point Likert scale (strongly disagree = 1 to strongly agree = 5), with α = 0.74. Perceived working conditions had 12 items measured on a 5-point Likert scale (strongly disagree = 1 to strongly agree = 5), with α = 0.65. Perceived job characteristics were measured using a 13-item questionnaire on a 5-point Likert scale (never = 1 to always = 5), with α = 0.75.

### Data collection and statistical analysis

The selected employees were contacted in their various hospitals. Questionnaires were given to each employee for completion after signing the consent for participation form. Dates for submission of questionnaires were agreed upon between authors and respondents. Completed questionnaires were collected from respondents by authors and research assistants. Questionnaires were administered between February and April 2019. Data were processed using the Statistical Package for Social Sciences (SPSS version 23.0). In all, 210 questionnaires were received and 31 were rejected for non-completion.

Descriptive statistics were employed to analyze the demographic data on participants and were presented using frequencies and percentages. Questionnaire items on the elements of the work environment of hospital employees and patient-centered behavior were aggregated into continuous variables by calculating the mean response across each of the five independent variables and the dependent variable using SPSS. The dependent variable (Patient-centered behavior) was tested for normality using a histogram. The data collected were analyzed to determine the effect of perceived work environment on patient-centered behavior based on a simple linear regression model. Stepwise multiple regression was conducted to establish a good prediction equation of patient-centered behavior of employees in the study.

### Ethical concerns

Written permission was obtained from the management of the hospitals included in the study to enable authors to collect data from their employees. Selected employees were contacted, and the purpose of the study and objectives were explained before participants could participate in the study. Ethical approval was obtained from the Ghana Health Service Ethics Review Committee, Research Division. Review No. GHS-ERC009/11/18. Additionally, Data were analyzed anonymously to ensure that the results cannot be traced to any of the respondents.

## Results

Table 1 presents sociodemographic information of respondents. Out of 270 questionnaires given out to selected hospital employees, 179 were fully completed and returned to authors, representing a response rate of 66%. The mean age of respondents was 30±4 years. The majority of respondents were between 20–30 years. A greater number (65.4%) of the respondents were females and Ghanaians (97.8%). Most (69.3%) of the respondents were clinical staff. Approximately 83.8% of the respondents indicated that they knew something about PCC.

**Table 1:** Descriptive statistics of respondents’ demographic background (N = 179).

Description	Freq.	%	Description	Freq.	%
***Sex***			***Age***		
Male	62	34.6	20–30	117	65
Female	117	65.4	31–40	52	29
***Nationality***			41–50	6	4
Ghanaian	175	97.8	50+	4	2
Non-Ghanaian	4	2.2	Mean age	30± 4	
***Relationship status***					
Single	94	52.5	***No*. *of Working Years***		
Married	83	46.4	1–10	156	87
Widowed	1	6	11–20	19	10
Divorce	1	6	21–30	3	2
***Religion***			40+	1	1
Christian	174	97.2	***Have Knowledge of PCC***		
Muslim	5	2.8	Yes	150	83.8
***Staff Category***			No	29	16.2
Clinical	124	69.3			
support service	55	30.7			

Source: computed by authors using field data.

Table 2 presents descriptive statistics of the patient-centered behavior of hospital employees. Most employees in the study admitted to often paying attention to patients’ needs (51%), respecting patients’ preferences and rights (52%), informing and educating patients (60%), and encouraging patients and allaying their fears (59%). All items on patient-centered behavior had a median score of 4.0.

**Table 2:** Descriptive statistics of patient-centered behavior of hospital employees (N = 179).

Behavior		frequency					
	Never	Seldom	Some times	Often	Very often	Total	Median
Involve patients in decisions regarding their care	1(1%)	11(6%)	23(13%)	92(51%)	52(29%)	179	4
Pay good attention to patients’ needs.	3(1.6%)	3(2%)	25(14%)	91(51%)	57(31%)	179	4
Respect the preferences and rights of patients.	4(2.2%)	11(6.2%)	40(22.3%)	93(52%)	31(17.3%)	179	4
Inform and educate patients about their health.	2(1.1%)	1(1%)	16(8.9%)	107(60%)	53(29%)	179	4
Comfort patients irrespective of condition.	0(0%)	8(4.4%)	24(13.4%)	107(60%)	40(22.2%)	179	4
Encourage patients and allay their fears.	5(2.7%)	11(6.2%)	21(12%)	106(59%)	36(20.1%)	179	4
Provide prompt response to patients’ complaints and questions.	2(1.1%)	7(4%)	28(16%)	103(57.3%)	39(21.7%)	179	4
Put patients first in all I do in this hospital.	3(1.6%)	3(1.6%)	22(12.3%)	85(47.5%)	66(37%)	179	4

Source: computed by authors using field data.

Tables 3 and 4 presents simple linear regression results on the effect of perceived work environment on the patient-centered behavior of employees of the hospitals. The adjusted r^2^ for perceived working conditions (Adj. r^2^ = 0.029), internal communication of PCC strategies and goals (Adj. r^2^ = 0.045), and supervisory support (Adj. r^2^ = 0.095) had a minimal positive effect on patient-centered behavior of employees of the hospitals in the study. Only perceived co-worker support had a moderate effect (Adj. r^2^ = 0.24) on patient-centered behavior. The regression ANOVA showed that the regression model for perceived co-worker support [F(1,177) = 58.97, P<0.001, r^2^ = 0.25], supervisory support [F(1,177) = 19.62, P<0.001, r^2^ = 0.10], internal communication of PCC strategies and goals [F(1,177) = 10.23, P<0.002, r^2^ = 0.06] and working conditions [F(1,177) = 6.27, P<0.013, r^2^ = 0.03] were statistically significant in predicting the influence of work environment on patient-centered behavior of employees of the hospitals included in the study.

**Table 3:** Model summary and ANOVA for regression on the effect of perceived work environment on patient-centered behavior.

	*R*	*R*^*2*^	*Adjusted R*^*2*^	*SE*	*Df*	*F*	*Sig*.
**Communication of PCC**					1	10.230	0.002
	0.234	0.055	0.049	0.52850	177		
					178		
**Supervisor Support**					1	19.619	0.001
	0.316	0.100	0.095	0.51572	177		
					178		
**Coworker Support**					1	58.968	0.001
	0.500	0.250	0.246	0.47076	177		
					178		
**Working Conditions**					1	6.270	0.013
	0.185	0.034	0.029	0.53417	177		
					178		
**Job characteristics**					1	3.765	0.054
	0.145	0.021	0.015	0.53902	176		
					177		

Source: computed by authors using field data. Significant at 0.05.

**Table 4:** Effect of perceived work environment on patient-centered behavior of hospital employees.

**Coefficients**						
	Unstandardized Coefficients		Standardized Coefficients	t	Sig.	Mean
	Beta	Std. Error	Beta			
(Constant)	3.484	0.179		20.852	0.000	
Communication on PCC	0.164	0.051	0.234	3.198	0.002	3.17
***Dependent variable*: *Patient-centered Behavior***						
**Coefficients**						
	Unstandardized Coefficients		Standardized Coefficients	t	Sig.	Mean
	B	Std. Error	Beta			
(Constant)	2.976	0.235		12.657	0.000	4.30
Supervisor Support	0.275	0.062	0.316	4.429	0.001	
***Dependent variable*: *Patient-centered Behavior***						
**Coefficients**						
	Unstandardized Coefficients		Standardized Coefficients	t	Sig.	Mean
	B	Std. Error	Beta			
(Constant)	1.419	0.338		4.191	0.000	
Coworker support	0.665	0.087	0.500	7.679	0.001	4.10
***Dependent variable*: *Patient-centered Behavior***						
**Coefficients**						
	Unstandardized Coefficients		Standardized Coefficients	t	Sig.	Mean
	B	Std. Error	Beta			
(Constant)	1.341	0.268		12.485	0.000	
Working Conditions	0.215	0.086	0.185	2.405	0.013	3.10
***Dependent variable*: *Patient-centered Behavior***						
**Coefficients**						
	Unstandardized Coefficients		Standardized Coefficients	t	Sig.	Mean
	B	Std. Error	Beta			
(Constant)	3.562	0.232		15.355	0.000	
Job Characteristics	0.150	0.077	0.145	1.940	0.054	2.95
***Dependent variable*: *Patient-centered Behavior***						

Source: computed by authors using field data. Significant at 0.05.

However, perceived job characteristics [F(1,176) = 1.094, P>0.054, r^2^ = 0.02] was not significant in predicting employees’ patient-centered behavior. The regression coefficients suggest a significant positive effect of perceived internal communication of PCC strategies and goals (β = 0.23; P<0.001) on the patient-centered behavior of employees; suggesting that if hospitals in the study improve internal communication of PCC strategies and goals from the current infrequent pattern to a regular pattern, patient-centered behavior would increase by 23%. Perceived supervisory support (β = 0.31; P<0.001), co-worker support (β = 0.50; P<0.001), and working conditions (β = 0.18; P<0.013) had a positive significant effect on patient-centered behavior of employees in the hospitals. This indicates that a unit increase in supervisory support and co-worker support would yield a 31% and 50% increase in patient-centered behavior for perceived supervisory and coworker support respectively. Perceived Job characteristics (β = 0.14; P>0.054) had no significant effect on the patient-centered behavior of employees.

Based on the regression results, H_1_, H_3_, H_4,_ and H_5_ were confirmed. We, therefore, conclude based on the results that, hospital employees’ perception of internal communication of PCC strategies and goals, supervisory support, co-worker support, and working conditions individually influence patient-centered behavior of employees of the hospitals in the study. However, H_2_ was not supported by the data. We conclude based on the regression output that perceived job characteristics do not affect the patient-centered behavior of employees of the hospitals included in the study.

Tables 5 and 6 present the results of the stepwise multiple regression of the elements of work environment that best predict patient-centered behavior of the hospital employees. Perceived co-worker support (Adj. r^2^ = 0.25) and job characteristics (Adj. r^2^ = 0.27) emerged as the strongest elements of the work environment for predicting patient-centered behavior of employees of the hospitals in the study. The stepwise multiple regression ANOVA models showed that perceived co-worker support [F(1,176) = 60.51, P<0.001, r^2^ = 0.26], and job characteristics [F(2,175) = 34.56, P<0.001, r^2^ = 0.28] were the strongest predictors of hospital employees’ patient-centered behavior. Perceived co-worker support (β = 0.51; P<0.001) and job characteristics (β = 0.16; P<0.011) had a significant positive effect on the patient-centered behavior of employees in the study. This indicates that 51% of employees’ patient-centered behavior could be explained by coworker support and 16% by job characteristics.

**Table 5:** Best predicters of patient-centered behavior of employees.

Model		Unstandardized Coefficients		Standardized Coefficients	T	Sig.	Correlations				
		B	Std. Error	Beta			Zero-order	Partial	Part.	Shared cont.	Unique cont.
1	(Constant)	1.381	0.339		4.072	0.000					
	Coworker Support	0.676	0.087	0.506	7.779	0.000	0.506	0.506	0.506		
2	(Constant)	0.841	0.394		2.133	0.034					
	Coworker Support	0.685	0.086	0.512	8.000	0.000	0.506	0.517	0.512	0.267	0.262
	Job Characteristics	0.172	0.066	0.165	2.580	0.011	0.145	0.191	0.165	0.036	0.027

Source: computed by authors using field data. Significant at 0.05.

**Table 6:** Model summary and AVOVA for good predictors of patient-centered behavior of hospital employees.

MODEL SUMMARY						ANOVA		
Model	R	R^2^	Adjusted R^2^	Std. Error	Durbin-Watson	Df	F	Sig.
1	0.506	0.256	0.252	0.46993		1	60.514	0.001
						176		
						177		
2	0.532	0.283	0.275	0.46256	1.973	2	34.557	0.001
						175		
						177		

Source: computed by authors using field data. Significant at 0.05

It should be noted that perceived job characteristics was significant in the stepwise regression analysis due to interactions between perceived job characteristics and co-worker support. Perceived co-worker support had a shared contribution of 26.7% and a unique contribution of 26% to employees’ patient-centered behavior. On the other hand, perceived job characteristics had a shared contribution of 3.6% and a unique contribution of 2.7% to employees’ patient-centered behavior, suggesting that the contribution of job characteristics to patient-centered behavior is significant, yet very little. Based on the stepwise regression results, we established the following prediction equation of the patient-centered behavior of employees of hospitals in the study as Y = 0.685X_1_+0.172X_2_+0.841+Ɛ. Where Y = patient-centered behavior of employees and Ɛ = the error term.

## Discussion

This study estimated the effect of hospital employees’ perception of their work environment on patient-centered behavior. Employees’ perception of working conditions in the hospitals had a significant effect on patient-centered behavior. From the simple linear regression results, job characteristics was not significant in predicting patient-centered behavior. Yet, a unique observation was made of a reaction between perceived job characteristics and co-worker support. In the stepwise multiple regression, job characteristics was the second-best model for predicting the patient-centered behavior of employees in the study. This supports findings of research from a teaching hospital in Kenya which established a significant positive effect of task identity and autonomy on nurses’ performance [21]. Job characteristics is closely knit with job design. When jobs are designed in a manner that motivates employees, satisfaction in the workplace improves [40]. The positive relationship between perceived job characteristics and patient-centered behavior suggests that hospital managers who wish to improve PCC in their hospitals may consider improving job characteristics by introducing job schedule redesign strategies to minimize monotony and improve autonomy, and task identity among front-line health workers.

The interaction between job characteristics and co-worker support further suggests that PCC improvement initiatives that seek to rely on enhanced job characteristics without improvement in co-worker support are unlikely to achieve significant outcomes. Hospital employees who enjoy job autonomy, job feedback, and task identity as suggested by Turner and Lawrence [41], may not significantly exhibit patient-centered behavior if they do not receive the support of co-workers. In a study on family and patient-centered care, it was found that good working relationships between and among work teams and staff wellbeing programs facilitate patient and family-centered care [5]. In another study, social support was found to increase the communicative responsiveness of caregivers and facilitated the provision of humanistic care to clients [42]. Even though the avalanche of literature on job characteristics focuses on the contribution of job characteristics to job satisfaction and employee performance, our findings contribute to the PCC literature by illuminating our understanding of the contribution of job characteristics to the patient-centered behavior of hospital employees.

Employees’ perception of both co-workers and supervisory support in this study were significant predictors of patient-centered behavior. In both the simple linear regression and stepwise multiple regression, co-worker support emerged as the strongest predictor of the patient-centered behavior of hospital employees. This means that workplace social support can serve as a valuable resource to hospital managers for improving PCC and acculturation of health professionals to PCC. Social support is a coping strategy against occupational stress; a factor found to be associated with poor outcomes in the healthcare setting, particularly among clinical staff [43, 44]. In this study, the clinical staff constituted the majority (69%), as such, interaction with and support from co-workers might have provided them with a sense of control over their work. Hence, the strong contribution of co-worker support to the patient-centered behavior of employees.

PCC thrives on the effort and commitment of members at all levels in the healthcare system, from the strategic to the operation-level. Yet, in this study, 16.2% of employees surveyed said they did not know about PCC, implying that these employees have no facts about PCC much more to provide services in a patient-centered manner. It also suggests that internal communication of PCC strategies and training in the hospitals excluded certain categories of staff. PCC is integral to the healthcare delivery process, from the patient information unit through to finance and administration and clinical units. All employees must assimilate the PCC culture of the hospitals to advance patient-centered experience of care [11]. Nonetheless, the results indicate that patient-centered behavior is the general attitude of the hospital employees. This could be attributed to the age structure of employees. Almost all employees were below 45 years with the majority of employees between the ages of 20–30 years. Even though research reveals mixed findings on the impact of age on employee performance [45–47], the age of the respondents in this study might have contributed to the patient-centered behavior spectacle. However, furtherstudies to explore the relationship between age diversity and the patient-centered behavior of hospital employees may be necessary.

Perceived internal communication of PCC strategies and goals had a significant influence on the patient-centered behavior of employees. Although the adjusted r^2^ value was low, the result reinforces earlier qualitative findings that communication of PCC mission and strategies among hospital employees is an essential activity for improving PCC. One basic purpose of internal communication is to keep organizational members informed of policy changes, build organizational culture, and engage employees. Through internal communication, hospital leaders can engage employees to share PCC goals and strategies to build a PCC culture in hospitals. Even though the present study did not experiment with the effect of communication of PCC strategies and goals on employees’ patient-centered behavior, the result is consistent with the literature [2, 42], and other findings on internal communication and employees’ performance [28, 29].

### Study limitation

Patient-centered behavior was assessed from employees’ perspectives and has the potential to generate inaccurate and insincere responses. This could partially be the reason behind the majority of employees indicating that they often behaved in a patient-centered manner. The study response rate of 66% has the potential to create false-positive findings. The work environment of hospital employees has elements that are linked to the emotions of employees. However, the use of self-reported questionnaires with Likert items may not have adequately captured the expressions and reactions of employees as would have been the case in a face-to-face interview scenario. The study examined the effect of the work environment of hospital employees and patient-centered behavior. Yet, the mediating effect of patient-centered behavior of hospital employees on the patient-centered experience of care is important in emphasizing the role of improved workplace environment and PCC. Further studies are required in this regard.

## Conclusion

The study examined the effect of perceived work environment on patient-centered behavior. As proposed by the study hypotheses, hospital employees’ perception of internal communication of PCC strategies and goals, supervisory support, co-worker support, and working conditions had a statistically significant effect on the patient-centered behavior of employees. The study also established that co-worker support, and job characteristics, in combination were the best predictors of the patient-centered behavior of employees in the study. Improving job characteristics alone as a strategy to improve patient-centered behavior of hospital employees may not be a worthwhile cause. Hospitals Managers seeking to improve patient-centered behavior through employee value creation may consider improved job characteristics in combination with workplace social support and or communication of PCC strategies and goals.

## Supporting information

S1 File (ZIP)

S2 File (SAV)
