# Supplementary material for: Perceived work environment and patient-centered behavior: A study of selected district hospitals in the central region of Ghana
Source: PLoS One. 2021 Jan 25;16(1):e0244726. doi: 10.1371/journal.pone.0244726 (PMC7833094; doi:10.1371/journal.pone.0244726)
Supplement: S1 File — (ZIP) [file pone.0244726.s001.zip › INSTRUMENT/JOB CHARACTERISTICS.pdf]

International Journal of Academic Research in Business and Social Sciences
2017, Vol. 7, No. 9
ISSN: 2222-6990

Effects of Job Characteristics on Employee Satisfaction in the Public Radio Stations in Syria

Abdul Hamid al Khalil
Department of Human Resource Management, Higher Institute of Business Administration, Damascus, Syria.

DOI: 10.6007/IJARBSS/v7-i9/3325 URL: http://dx.doi.org/10.6007/IJARBSS/v7-i9/3325

ABSTRACT
Based on the existing literature indicates that job characteristics, job satisfaction, organizational commitment, and organizational citizenship behavior are very important for effective organizational functioning, the present study has predicted the job satisfaction level of the general radio stations employees through job characteristics by investigated a complete relationship between five job characteristics as antecedent of job satisfaction.
The primary data was collected through the job diagnostic survey originally developed by Hackman & Oldham (1975) from (289) employees and from different sections, with the help of Regression Analysis by using SPSS we have found a positive and significant relationship among various job characteristics and job satisfaction andrevealed that job characteristics has been significantly affecting job satisfaction.
Keywords: Job characteristics, Job Satisfaction.

INTRODUCTION

Much of the history of management and motivation theory is rooted in the desire to understand the factors that contribute to increased levels of job performance and workplace productivity. Not surprisingly, ratings of job satisfaction have consistently served as one of the highest correlates of job performance and productivity (Abdullah, 2004). And moreoverjob satisfaction has been the most widely studied conception in the history of industrial/organizational psychology (Judge et al., 2002).
The degree of job satisfaction and job performance are correlated with each other and whenever the job satisfaction level is higher it can be due to various other sub reasons (Chang & lee, 2006)
Also the concept of job characteristics model that focus on designing jobs to motivate people and to make them more satisfy on their workplaces (Droar, 2006).
The theoretical background of this research has its origin in job design. During people working in the organizations has been performing daily a lot of activities and if they are allowed to perform according to their skills, abilities, and knowledge then they are self-motivated to perform at their best.
In order to create high Job satisfaction all elements of job characteristics must exist in work settings this was the basic premise of Job characteristic theory also it helpful in reducing employee turnover and absenteeism, so Hackam and Oldham (1980) measured how job

characteristics flares up employee satisfaction, enthusiasm, and performance and found interest results (Fried & Ferris,1987)

Research problem

Based on the significant contribution of the nature of any job on employees satisfaction at work as one of many other variables which predict job satisfaction (like pay, supervisor …) and based on need to translate this nature by many dimensions we will test the job characteristics as recommended by Hackman and Oldham to explore the ability of them to explain the role of job nature on job satisfaction.
Upon we can ask later questions.

Research questions

1. Does Skill variety predict job satisfaction?
2. Does Task Identity predict job satisfaction?
3. Does Task significance predict job satisfaction?
4. Does Autonomy predict job satisfaction?
5. Does Feedback predict job satisfaction?

Research Objectives

The purpose of the study was to examine the effects of job characteristics on employee satisfaction in audio able public media sector. The research study was guided by the following objectives.
i. To examine the influence of job autonomy on employee job satisfaction in the audio able public media sector in Syria.
ii. To establish the effects of task significance on employee job satisfaction in the audio able public media sector in Syria.
iii. To assess the effects of skills variety on employee job satisfaction in the audio able public media sector in Syria.
iv. To investigate the influence of feedback from job on job employee satisfaction in the audio able public media sector in Syria.
V. To assess the effects of task identity on employee job satisfaction in the audioable public media sector in Syria.

Literature review

There are many theories of job satisfaction like the theory of Herzberg that used and studied widely by scholars to explain person what does he/she wants during working in the organization.(Chang & lee, 2006)
Job design refers to the way tasks are combined to form complete jobs and the process of job design has evolved over a long period of time. For the first time the operational measures of the job characteristics were given by Turner & Lawrence (1965). They developed six task attributes assumed to be positively related to workers satisfaction and attendance. The results revealed close relationship among variables and on the basis of results they developed required

task attribute index. On the basis of this summary index relationship between task attributes and job satisfaction and attendance was determined. The results were not fully supported. (Fried & Ferris, 1987).

Based on it job diagnostic survey, Hackman, Oldham (1976) developed this approach. Job characteristics are objective characteristics of jobs, particularly the degree to which jobs are designed so that they enhance the internal work motivation and the job satisfaction of job incumbents. The five core job features, which are task significance, task variety, task identity, autonomy and feedback, believed to positively correlate with job satisfaction and performance (Spector & Jex 1991).

Job satisfaction theory can divide in to two catalogs; first, job content satisfaction refers to a factor that makes personnel satisfied and the motivation to perform such as achievement, recognition, responsibility, advance, and work itself. Second, the job context which refers to factors external components preventing job dissatisfaction, such as company policy and administration, supervision, relationship with peers, salary and benefit, security, and working condition.(Champoux, 1991).

A lot of previous empirical studies that focus on job characteristics havefound a substantial influence on job satisfaction. (Judge, 2001)and these correlationsalso confirmed between objective job characteristics and job satisfactionamong school teachers in Colombo districtwhen their growth need was high(Bhuain, et al., 2001).

Also (Smits et al. 1993)confirmed in their study that job characteristics and individual characteristics are related to job satisfaction.

(Hackamn & Oldham, 1976) suggested that a job characteristic, such as skill variety, Task identity, autonomy, and feedback brings motivation in employees about their job performance.

The Concepts of the study

Job Characteristics

Hackman & Oldham (1975) empirically tested the relationships among the job characteristics, individual differences in need strength and employee's motivation, satisfaction, performance, and absenteeism on the job. The results indicated that positive relationship was found among job dimensions and dependent measures: motivation, satisfaction, performance and attendance.

It is especially suitable for those who have the strong desire for feeling of accomplishment and growth. Individuals who are low on growth need strength may find such job difficult to perform and may feel uncomfortable with it.

At (1976) Hackman & Oldham empirically tested the relationships postulated by JCM including the mediating effect of CPS's and moderating effect of GNS. In general all correlations between JC, PS and outcomes (except absenteeism) were positive and negative for absenteeism as expected. The mediating effect of CPS was proved through the partial correlations and multiple regressions. The moderating role of GNS was also proved by determining separate correlations for employees high and low on GNS measurement scale. The correlation results were higher for those who were high on GNS as compared to those who were low on GNS.

Based on their model there are five core dimensions of job characteristics.

- Skill Variety is the degree to which a job requires a variety of different activities in carrying out the work, which involve the use of a number of different skills and talents of the employee.
- Task Identity is the degree to which the job requiresthe completion of a “whole” and identifiable piece ofwork (i.e.; doing a job from beginning to end with visible outcomes).
- Task Significance is the degree to which a job has asubstantial impact on the lives or work of otherpeople whether in the immediate organization or in theexternal environment.
- Autonomy is the degree to which the job providessubstantial freedom, independence, and discretion tothe employee in scheduling his or her work and in determining the procedures to be used in carrying itout.
- Feedback refers to the degree to which carrying outthe work activities required by the job results in theemployee obtaining information about the effectivenessof his or her performance.

Job satisfaction

The concept of job satisfaction has been described as a multifaceted response that involves emotional and behavioral expression. Additionally, job satisfaction is an affective reaction between the actual work accomplished and the desired outcome (Hazem, et al., 1999)

Job satisfaction refers to the attitude of personnel concern with job which is the result of perception of job performing and the level of cohesion between individual and organization (Smits, et al.,1993)

Hypothesizes:

Hypothesis 1: There is a positive and significant relationship between Skill Variety andjob satisfaction.

Hypothesis 2: There is a positive and significant relationship between Task Identity andjobsatisfaction.

Hypothesis 3: There is a positive and significant relationship between Task Significance and jobsatisfaction.

Hypothesis 4: There is a positive and significant relationship between Autonomy and job satisfaction.

Hypothesis 5: There is a positive and significant relationship between Feedback and jobsatisfaction.

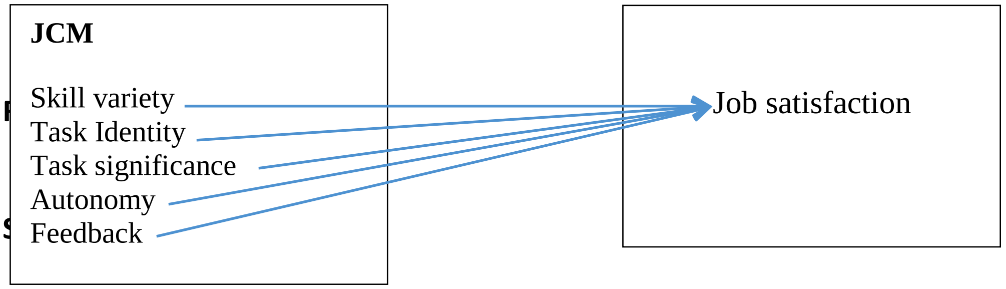

The data was collected from the employees of 4public radio stations. The sample consisted of 289employees.

Instruments:

<u>Job diagnostic survey (JDS)</u>

Hackamn and Oldham (1976) developed a self-report instrument to measure the five core job features of job characteristics model. The questionnaire consists of 18 items, and items were scored on 5 point Likert type scale.

<u>Job Satisfaction Survey:</u>

A Questionnaire of job satisfaction was used by (**Weiss**at al., 1999)consisting of 20 Items Rated on 5 point Likert type scale to measure job satisfaction..

Analysis Technique:

SPSS software was used to analyze the questionnaires a total sample of 289employees is selected and from this an equal number of questionnaires have been filled up.

Demographics	N	%
**Gender**		
Male	139	48
Female	150	52
**Age**		
25 and below	38	13
21-30	163	56
31-40	53	18
Above 40	35	13
**Education**		
High school	66	23
Institute	76	26
University	156	51

Table 1

From the table 1 we can notice that our sample is diver and moderate between males and females otherwise the higher stage of age is between 21 – 30 years that introduce better result because this stage is more energy and able to talk about their jobs and diagnosis its.

And finally the level of education is acceptable as we can see from the table more than half of the sample is academic personsthat lead to more realistic data and result

Test of hypotheses:

The hypotheses were tested by using the multiple regression analysis from SPSS software program and the results are showed in table 2

Table 2: means, standard deviations, and correlations

International Journal of Academic Research in Business and Social Sciences
2017, Vol. 7, No. 9
ISSN: 2222-6990

	mean	SD	Skill variety	Task significance	Task identity	Autonomy	Feedback	Job satisfaction
Skill variety	3.42	1.24	(86)					
Task significance	3.77	1.08	.23**	(77)				
Task identity	2.98	1.10	.25*	.23**	(75)			
Autonomy	3.33	1.13	.15**	.18**	.19**	(82)		
Feedback	3.88	1.31	.23**	.19*	.21*	.19**	(77)	
Job satisfaction	3.55	0.95	.28**	.25**	.21**	.28**	.22**	(73)

Note. *p <05, **p<01 Table 2

Note. * =>p <05, **=>p<01

Based on the table 2 all alpha cronbach rates are more than 70% that means all are acceptable and the internal accordance will be high.

For means and SD we can notice all of them are acceptable whereas significance and strength of the relations we will discuss it in the results.

Based on the reference data from Hackman and Oldham the current jobs are acceptable and after comparing it's we can say that most jobs need reengineering or first we recommendedestimating the current tools that used then we can reevaluate the fivecore dimensions of job characteristics.

Hypothesis	result
Hypothesis 1: There is a positive and significant relationship between Skill Variety andjob satisfaction.	accepted
Hypothesis 2: There is a positive and significant relationship between Task Identity andjobsatisfaction.	accepted
Hypothesis 3: There is a positive and significant relationship between Task Significance and jobsatisfaction.	accepted
Hypothesis 4: There is a positive and significant relationship between Autonomy and jobsatisfaction	accepted
Hypothesis 5: There is a positive and significant relationship between Feedback and jobsatisfaction	accepted

Table 3

Results:

Based on the table 3 there is a significant relationship at the (.01) confidence level between Skill Variety andjob satisfaction.

Prior studies found a positive relationship between Skill Variety and job satisfaction (Droar, 2006, Bhuain et al., 2001).

For instance, in their study of sales representatives, (Abdullah, 2004) found that individuals who scored high on Skill Variety were more likely to set and be committed to goals, which in turn translated to a positive relationship between Skill Variety and job satisfaction
Based on the table 3 there is a significant relationship at the (.01) confidence level between Task Identity andjob satisfaction

Empirical evidence shows a positive relationship between Task Identity andjob satisfaction(Chang &Lee, 2006).
Also (Griffeth et al. 2000) reported a positive relationship between Task Identity andjob satisfaction
Based on the table 3 there is a significant relationship at the (.01) confidence level between Task Significance and job satisfaction.
(Humphrey et al.,2007) noted that few studies have examined the association between Task Significance and job satisfaction also(Saavedra, & Kwun, S. K.2000) Found a positive relationship between Task Significanceand job satisfaction
Based on the table 3 there is a significant relationship at the (.01) confidence level between Autonomy and job satisfaction
The positive link between Autonomy and job satisfaction of employees also founded with past studies (Champoux, 1991).But with others there is no significance relationship between autonomy and job satisfaction. (Smits, et al.,1993)

Based on the table 3 there is a significant relationship at the (.01) confidence level between Feedback and job satisfaction.
(Hillol & Viswanath, 2013) concluded that there was a positive correlation between Feedback and job satisfaction, but (Spector & Jex, 1991) study determined that Feedback was a valid predictor of job satisfaction.

Limitations and future research
There are several limitations of this study that can be addressed in future research:
First, the demographics of this study limit the external reliability of the findings specifically due to the uniqueness of the public radio stations in Syria.
Second, employee in public radio stations in Syria may have different working conditions than other employee in other places either in the same job or other sector.
Second, employee in public radio stations in Syria may have different working conditions than other employee in other places either in the same job or other sector.
Third, job satisfaction must necessarily be studied using self-reports, non-self-report measures of objective and subjective satisfaction that would have enhanced the validity of the findings.
Finally, factors such as years of experience and employee benefits were not considered for the very important impact of these factors in generating the idea of leave in comparative with other places.
Future studies should test the relationships with many factors such as organizational, personal, occupational and cultural elements apart from job characteristics, demographic variable and

role ambiguity that may influence turnover intentions and should be explored for further research.

Conclusion:
These findings in this study present a very important contribution in explain the effect of the nature of job on levels of satisfaction with different rates based on the ability of each dimension of job, but in general we can say by increasing the all job characteristics we will achieve more satisfaction levels.

JobCharacteristics					
**Items**	1	2	3	4	5
**Skill variety**					
I get to use a number of complex skills on this job.					
The job involves doing a number of different tasks.					
I can use the skills that I have to handle the problem in my job.					
I have chance to using a wide variety of different skills and talents.					
**Task identity**					
I do a complete task from start to finish.					
I have chance to do an entire piece of work from beginning to end.					
Once I start a task I have to finish it.					
I never work just do half and then assign to others.					
**Task significance**					
Only when my job gets done, other jobs can be completed.					
My job is always to get the attention of co-workers.					
My job is an integral part of the organization.					
**Job Autonomy**					
I control the content of my job.					
I have freedom to decide how I perform assigned tasks.					
I can set my own schedule for completing assigned tasks.					
**Job Feedback**					
The work shows me with information about my performance.					

Supervisor provides me with constant feedback about my activity.					
I am clearly competent view of my work performance					
I can timely know whether my job is efficiency.					

**Job satisfaction**					
Items	1	2	3	4	5
I am satisfied with the working environment of the company.					
I am satisfied with job location.					
I am satisfied with the present working hour.					
I am satisfied with the existing salary structure of the company.					
I am satisfied with the compensation I get & I think it matches with my responsibility.					
I am happy with my work responsibilities.					
I feel comfortable in carrying out my responsibilities.					
I am satisfied with work relationships with the people around me.					
I am satisfied with various activities in the firm & love participating in them.					
I am happy with your overall job security.					
I am satisfied with the given right to put forward my opinions.					
I am satisfied with the leaders in my workplace as positive role models.					
I am satisfied with the present performance appraisal policy of the company.					
I am happy with the recognition and rewards for my outstanding works and contributions.					
I am satisfied and think I've been awarded right set of duties, as per my ability.					
I am satisfied & able to maintain a healthy balance between work and family life.					
Fulfilling my responsibilities give me a feeling of satisfaction & personal achievement.					
I am satisfied with the leave policy of the company.					
I am satisfied employee assistance policy					

(e.g.- lunch & transport etc.) of the company.					
I am satisfied with long term benefit & insurance policies of the company					

References

- Abdullah, N. N. (2004). Job satisfaction in relation to work motivation among sales representatives. Unpublished masters dissertation, Quaid-e-Azam University, Islamabad.
- Bhuain, S. N., Al-Shammari, E. S., & Jefri, O. A. (2001). Work-related attitudes and job characteristics of expatriates in Saudi Arabia. Thunderbird International Business Review, 43(1), 21-31.
- Brief, A.P., Butcher, A.H., & Roberson, L. (1995). Cookies, disposition and job attitudes: The effects of positive mood-inducing events and negative affectivity on job satisfaction in a field experiment. Organizational Behavior & Human Decision Processes, 62(1), 55-62.
- Champoux, J.E. (1991). A multivariate test on the job characteristics theory of work motivation. Journal of Organizational Behavior, 12(5), 431-446
- Chang, C. S., & Lee, M. S. (2006). Relationships among personality traits, job characteristics, and organizational commitment: An empirical study in Taiwan. The Business Review, Cambridge, 6(1), 201-207.
- Droar, D. (2006). The Job characteristics model. Retrieved May 1, 2009, from characteristics .php.
- Fried, Y., & Ferris, G. R. (1987). The validity of the job characteristics model: a review and meta-analysis. Personnel Psychology, 40, 287 – 322.
- Gattiker, U. E. & Larwood, L. (1989). Career Success, Mobility and Extrinsic Satisfaction of Corporate Managers," Social Science Journal, 26, 75-92.
- Griffeth, R. W., Horn, P. W., & Gaertner, S. (2000). A meta-analysis of antecedents and correlates of employee turnover: Update, moderator tests, and research implications for the next millennium. Journal of Management, 26(3), 463-488.
- Hackman, J. R. (1976). Work redesign. In J. R. Hackman & J. L. Shuttle (Eds.). Improving life at work (pp. 96-162). Santa Monica, CA: Goodyear.
- Hackman, J. R., & Oldham, G. (1975). Development of the job diagnostic survey. Journal of Applied Psychology, 60, 159-170.
- Hackman, J. R., & Oldham, G. R. (1976). Motivation through the design of work: Test of a theory. Organizational Behavior and Human Performance, 16, 250-279.
- Hackman, J. R., & Oldham, G. R. (1980). Work design in the organization context. Research in Organizational Behavior, ,2., 247-278. 130
- Humphrey, S. E., Nahrgang, J. D., & Morgeson, F. P. (2007). Integrating motivational, social, and contextual work design features: A meta-analytic summary and theoretical extension of the work design literature. Journal of Applied Psychology, 92(5), 1332-1356.

- Judge, T. A., Heller, D. & Mount, M. K. (2002). "Five-Factor Model of Personality and Job Satisfaction: a Meta-Analysis," Journal of Applied Psychology, 87(3), 530-541.
- Saavedra, & Kwun, S. K. (2000). Affective states in job characteristics theory. Journal of Organizational Behavior, 21, 131-146.
- Smits, S. J., Tanner, J. R., & McLean, E. R. (1993). Job Characteristic preference reality discrepancies and the job career attitudes of I/S professionals. Association for Computing Machinery, 120-130.
- Spector, P. E., & Jex, S. M. (1991). Relations of job characteristics from multiple data sources with employee affect, absence, turnover intentions, and health. Journal of Applied Psychology, 76, 46-53.
- Stamps, P. (1997). Nurses and work satisfaction: An index for measurement (2nd ed). Chicago: Health Administration Press.
- Weiss, D.J, Dawis, R.V., England, G.W., & Lofquist, L.H. (1967). Manual for the Minnesota Satisfaction Questionnaire. Minneapolis: University of Minnesota, Industrial Relations Center.
- Weiss, H.M., Nicholas, J.P., & Daus, C.S. (1999). An examination of the joint effects of affective experiences and job beliefs on job satisfaction and variations in affective experiences over time. Organizational Behavior and Human Decision Processes, 78(1), 1-24.
