# Supplementary material for: Perceived work environment and patient-centered behavior: A study of selected district hospitals in the central region of Ghana
Source: PLoS One. 2021 Jan 25;16(1):e0244726. doi: 10.1371/journal.pone.0244726 (PMC7833094; doi:10.1371/journal.pone.0244726)
Supplement: S1 File — (ZIP) [file pone.0244726.s001.zip › INSTRUMENT/RESEARCH INSTRUMENT.docx]

**PCC QUESTIONNAIRE**

**Preamble**

This instrument seeks to collect data from different categories of hospital staff to assess their work environment and patient-centered behavior of hospital employees in first-level referral hospitals in selected districts of the Central Region of Ghana. Data collected through this research will be used purely for academic work/publications. The anonymity of all respondents in the study is guaranteed. You are therefore requested to be as objective and realistic as possible when responding to the question below.

Questions are divided into three main sections. Section A solicits for background information of respondents. Section B assesses the patient-centered behavior of employees and section C assesses employees’ perceptions about their work environment.

**Section A: Background Information of Respondents**

| **Socio-Demographic/Work Background Respondents**  Please. provide us with some basic information about yourself and your work. | **Options** |
| --- | --- |
| Age (please, write your age in the next column). | [ ] |
| Gender | Male [ ]female [ ] |
| Nationality | Ghanaian [ ] Non-Ghanaian [ ] |
| Relationship status | Single [ ]Married [ ]  widowed [ ] Divorced [ ] |
| Religion | Christian [ ] Muslim [ ]  Others [ ] |
| Work experience/length of service | Years [ ]. |
| How long have you been working in this hospital | Years [ ] |
| What is your present position (please, indicate this in the next column ) |  |
| Staff category | Clinical [ ]. support Service [ ]. |
| How many hours do you work in your hospital? (please, indicate this in the next column ) |  |
| How do you commute from your house to the workplace | Walking [ ]. Biking [ ]. Car [ ]  Public transport [ ]. Others [ ] |
| Do you know anything about patient-centered care? | Yes [ ]. No [ ]. |

**Section B – Patient-centered Care Behavior**

| **Patient centered-care Behavior**  Consider the following, and indicate how frequent you do them? Please, check (√) as appropriate | Never  (1) | Seldom  (2) | Sometimes (3) | Often  (4) | Always  (5) |
| --- | --- | --- | --- | --- | --- |
| I involve patients and family in decision making regarding their care |  |  |  |  |  |
| I pay attention to patients’ needs |  |  |  |  |  |
| The patient is the center of everything I do in the hospital |  |  |  |  |  |
| I inform and educate patients on everything about their condition |  |  |  |  |  |
| I ensure that patients are comforted irrespective of their condition |  |  |  |  |  |
| I allay the fears of patients and reassure them |  |  |  |  |  |
| I provide patients with prompt services |  |  |  |  |  |
| I put the patient first in all I do in this hospital |  |  |  |  |  |

**Section D – Work Environment**

|  |  |  |  |  |  |
| --- | --- | --- | --- | --- | --- |
| **Internal communication on PCC values, strategic vision, and Goals**  Consider your working situation, how often do you experience the following situations | Never  (1) | Seldom  (2) | Some-times  (3) | Often  (4) | Every often  (5) |
| My hospital explains to me all I need to know about PCC |  |  |  |  |  |
| My unit head discusses with me my role in providing PCC in this hospital |  |  |  |  |  |
| I share with co-workers on how to improve PCC in this hospital |  |  |  |  |  |
| My hospital shares with all staff its strategic goals on PCC |  |  |  |  |  |
| My hospital display PCC strategies and goals at vantage points at in the hospital |  |  |  |  |  |
| My hospital hold staff meetings to discuss PCC improvements |  |  |  |  |  |
| My hospital constantly reminds me that my role is very important in achieving PCC goals |  |  |  |  |  |

| **Supervisory Support**  Consider your hospital, to which degree would you agree or disagree with the following statement Please, check (√) as appropriate. | Strongly disagree  (1) | Disagree  (2) | Neutral  (3) | Agree  (4) | Strongly  Agree  (5) |
| --- | --- | --- | --- | --- | --- |
| My unit head is always concern about meeting the needs of patients |  |  |  |  |  |
| My unit head has a good relationship with me |  |  |  |  |  |
| My unit head motivate me to give my best |  |  |  |  |  |
| I feel appreciated by my unit head |  |  |  |  |  |
| My unit head is a good role model |  |  |  |  |  |
| when I make suggestions to my unit head it is always taken seriously |  |  |  |  |  |
| My unit head always take action on the suggestion from me and my co-workers |  |  |  |  |  |
| My unit head is available when I need him/her |  |  |  |  |  |
| I feel comfortable to ask the unit head if I don’t understand what I am supposed to do |  |  |  |  |  |
| My unit head seldom give positive feedback |  |  |  |  |  |
| **Taking all the above factors into consideration how satisfied are you with your:** | Very dissatisfied (1) | Dissatisfied (2) | Neutral  (3) | Satisfied  (4) | Very Satisfied  (5) |
| Unit Head |  |  |  |  |  |
| **Co-worker Support**  Consider your hospital, to which degree would you agree or disagree with the following statement Please, check (√) as appropriate. | Strongly disagree  (1) | Disagree  (2) | Neutral  (3) | Agree  (4) | Strongly  Agree  (5) |
| My co-workers respect my opinion and personal values |  |  |  |  |  |
| I respect my co-worker’s opinion and personal values |  |  |  |  |  |
| I get along well with my co-workers |  |  |  |  |  |
| My co-workers share positive comments about each other |  |  |  |  |  |
| My co-workers accept my suggestions about work |  |  |  |  |  |
| My co-workers offer to help with my work when I need it |  |  |  |  |  |
| I carry out my responsibilities to the best of my ability |  |  |  |  |  |
| I respect my co-worker’s style of time management and priorities |  |  |  |  |  |
| I always adapt my behavior to avoid confrontation at the workplace |  |  |  |  |  |
| I always welcome new persons in my department |  |  |  |  |  |
| **Taking all the above factors into consideration how satisfied are you with your:** | Very dissatisfied (1) | Dissatisfied (2) | Neutral  (3) | Satisfied  (4) | Very Satisfied  (5) |
| Co-workers |  |  |  |  |  |

| **Working Conditions**  Consider your hospital, to which degree would you agree or disagree with the following statement? Please, check (√) as appropriate. | Strongly disagree  (1) | Disagree  (2) | Neutral  (3) | Agree  (4) | Strongly  Agree  (5) |
| --- | --- | --- | --- | --- | --- |
| My work unit supports my comfort |  |  |  |  |  |
| There are no disturbing noises at my work unit |  |  |  |  |  |
| Illumination and ventilation are working well in my work unit |  |  |  |  |  |
| My hospital supply almost all the materials and tools I need to work with |  |  |  |  |  |
| My role in this hospital is relevant |  |  |  |  |  |
| There is an opportunity for carrier advancement in this hospital |  |  |  |  |  |
| In this hospital, I am required to do the same task the same way every day |  |  |  |  |  |
| I am paid well for the work I do and the position I hold |  |  |  |  |  |
| My salary is the only reason I continue to work in this hospital |  |  |  |  |  |
| I will always be allowed time to attend to my family should something happen to my family |  |  |  |  |  |
| I am satisfied with the length of my annual leave period |  |  |  |  |  |
| I have great loyalty to this hospital |  |  |  |  |  |
| **Job Characteristics**  Considering your job characteristics, how often do the following happen? Please, check (√) as appropriate. | Never  (1) | Seldom  (2) | Some-times  (3) | Often  (4) | Every often  (5) |
| I made to perform several different tasks |  |  |  |  |  |
| I am allowed to use the skills I have to solve problems in my unit |  |  |  |  |  |
| The hospital revises my work schedules |  |  |  |  |  |
| I am made to attend to too many people at the same time |  |  |  |  |  |
| I am made to attend to clients even when it is time to close |  |  |  |  |  |
| I am assigned a task that I can start and finish |  |  |  |  |  |
| my work is an input into the work of my co-workers |  |  |  |  |  |
| My work gets the attention of co-workers and patients |  |  |  |  |  |
| I am made to believe that my work is important to this hospital |  |  |  |  |  |
| I take part in deciding what should be my job schedule |  |  |  |  |  |
| I follow strict procedures to carry out my work |  |  |  |  |  |
| My unit head appreciate the work I do |  |  |  |  |  |
| My unit head gives me feedback on my work |  |  |  |  |  |
